# Supplementary material for: Optimal acceleration voltage for near-atomic resolution imaging of layer-stacked 2D polymer thin films
Source: Nat Commun. 2022 Jul 8;13:3948. doi: 10.1038/s41467-022-31688-4 (PMC9270374; doi:10.1038/s41467-022-31688-4)
Supplement: Supplementary file 1 — Supplementary Information [file 41467_2022_31688_MOESM1_ESM.pdf]

Supplementary Information

Optimal acceleration voltage for near-atomic resolution imaging of layer-stacked 2D polymer thin films

Baokun Liang[1,†], Yingying Zhang[2,†], Christopher Leist[1], Zhaowei Ou[3], Miroslav Položij[2], Zhiyong Wang[2], David Mücke[1], Renhao Dong[2,4], Zhikun Zheng[3], Thomas Heine[2,5,6], Xinliang Feng[2,7], Ute Kaiser[1]*, Haoyuan Qi[1,2]*

Affiliations

[1]Central Facility for Electron Microscopy, Electron Microscopy Group of Materials Science, Universität Ulm, 89081 Ulm, Germany.

[2]Faculty of Chemistry and Food Chemistry & Center for Advancing Electronics Dresden (cfaed), Technische Universität Dresden, 01062 Dresden, Germany.

[3]Key Laboratory for Polymeric Composite and Functional Materials of Ministry of Education, School of Chemistry, and State Key Laboratory of Optoelectronic Materials and Technologies, Sun Yat-sen University, 510275 Guangzhou, P. R. China.

[4]Key Laboratory of Colloid and Interface Chemistry of the Ministry of Education, School of Chemistry and Chemical Engineering, Shandong University, 250100 Jinan, P. R. China.

[5]Helmholtz Center Dresden-Rossendorf, Institute of Research Ecology, Leipzig Research Branch, 04318 Leipzig, Germany.

[6]Department of Chemistry, Yonsei University, 03722 Seoul, Republic of Korea.

[7]Max Planck Institute of Microstructure Physics, 06120 Halle (Saale), Germany.

*Corresponding authors: haoyuan.qi@uni-ulm.de (H.Q.); ute.kaiser@uni-ulm.de (U.K.)

[†] These authors contributed equally to this work.

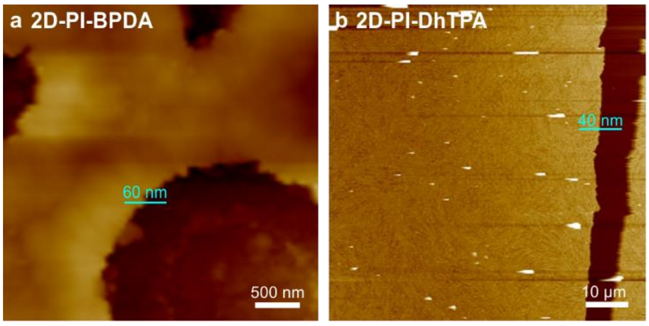

Fig. S1. AFM measurements of 2D-PI-BPDA and 2D-PI-DhTPA. The thickness of the 2D polymer thin films was measured at the film edges or at cracks.

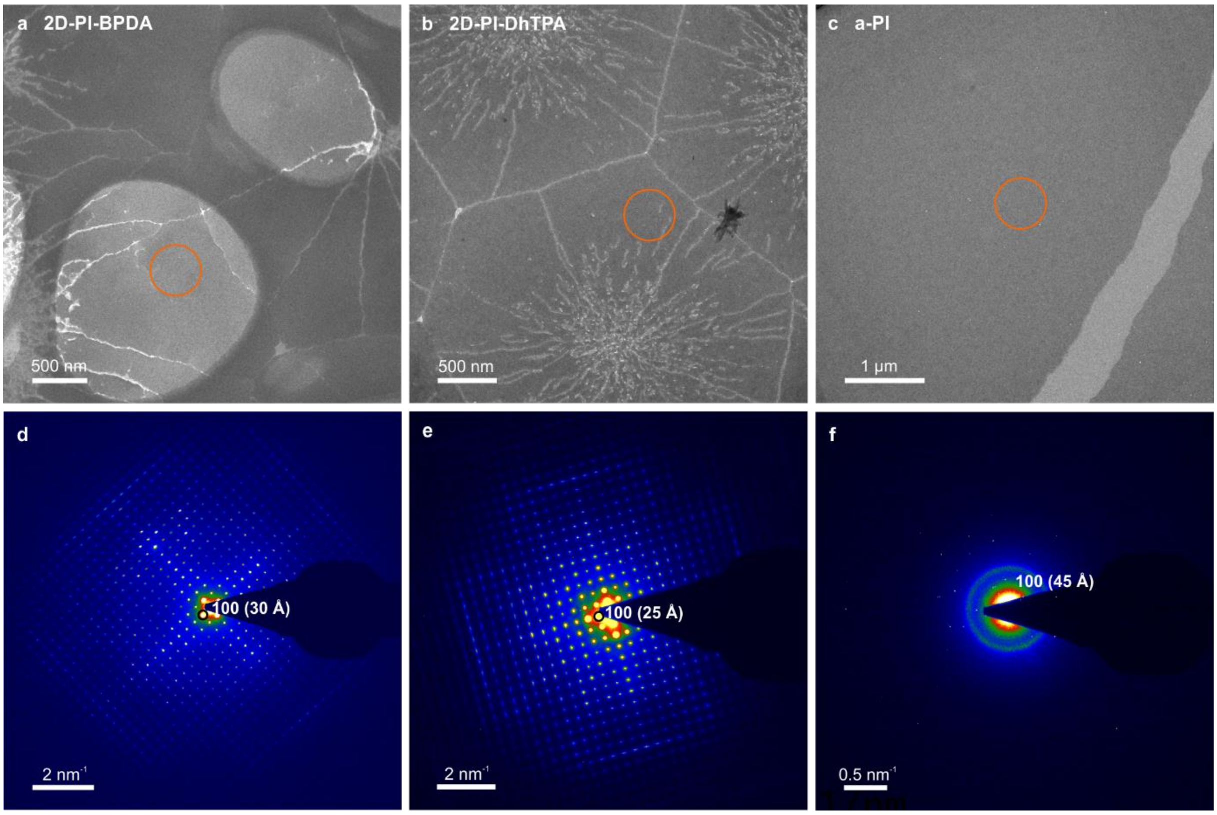

Fig. S2. Bright-field and SAED images of 2D-PI-BPDA, 2D-PI-DhTPA, and *a*-PI. a-c, Bright-field TEM images. The orange circles mark the position of the applied selected-area aperture for SAED pattern acquisition. The selected circular area has a diameter of ca. 480 nm. d-f, SAED patterns obtained from the circular region in a, b and c, respectively. The 100 reflections and the corresponding lattice distances are indicated.

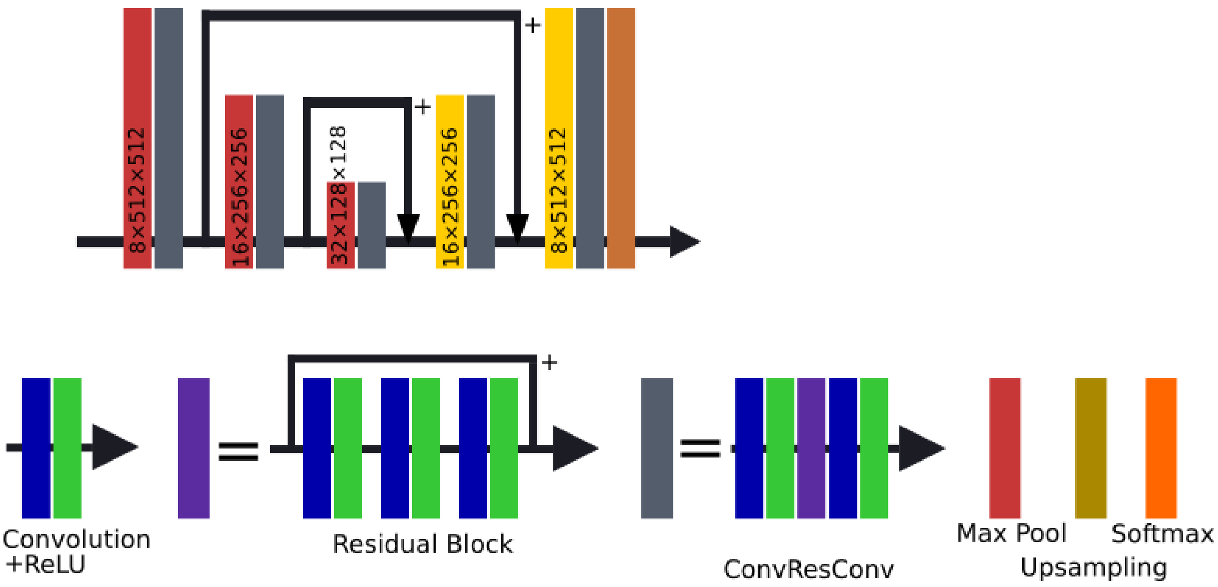

Fig. S3. Structure of the 2-step neural network. The neural network was trained for 300 epochs, each containing one batch of the five images, where the images are randomly rotated. To prevent interpolation errors in the rotated images, only 90-degree steps were used. Skip connections were employed to reintroduce information lost by the down-sampling steps. In the last layer of the network, a softmax-function was applied to generate a probability map. The position of the diffraction spots can then be extracted from this map.

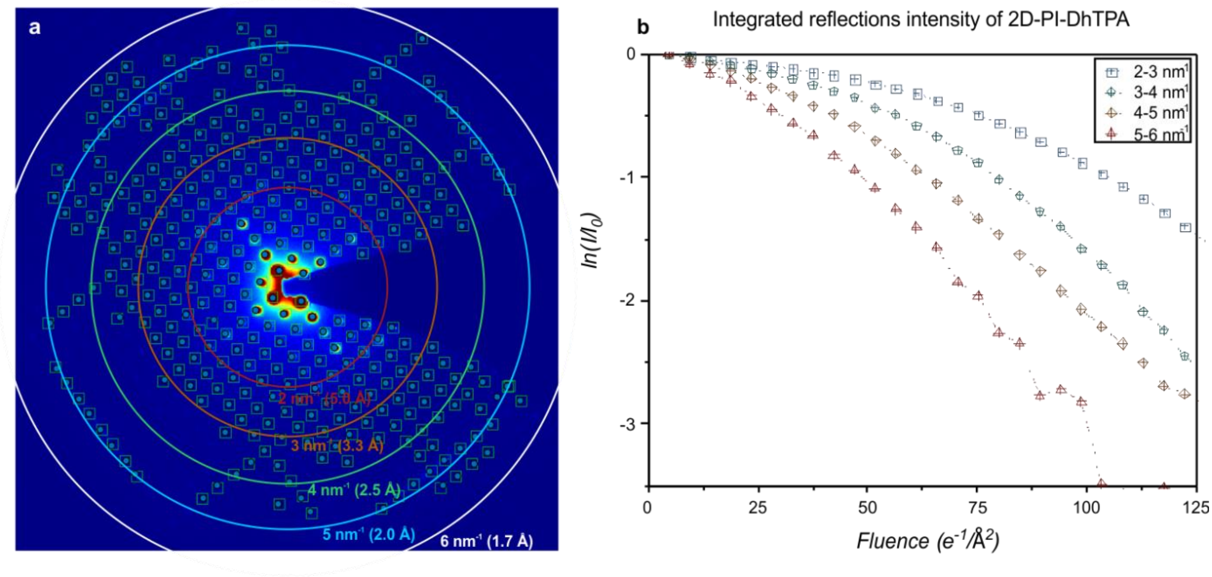

Fig. S4. Critical fluence determination method. a, One of the selected diffraction patterns from a SAED series on 2D-PI-DhTPA acquired with a constant electron flux (0.48 $e^-/Å^2s$). The rings mark the position of different spatial resolutions. b, Integrated intensity profile of the reflections inside each resolution range (2-3, 3-4, 4-5, 5-6 nm^{-1}) as a function of the electron fluence.

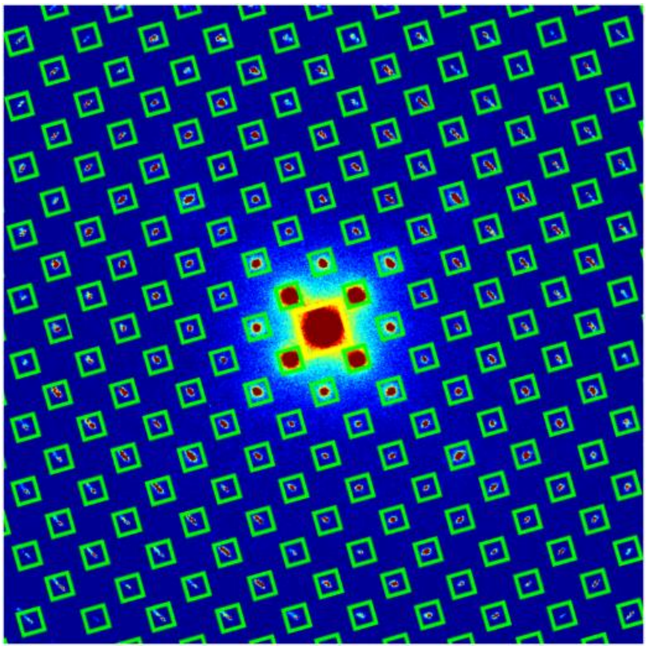

Fig. S5. Structural information determination. The SAED pattern is acquired without the beam stop. The central beam is marked by the yellow square and the diffracted beams with green boxes. The structural information is determined by the ratio of the integrated Bragg reflections intensity (I_{Bragg}) to the total intensity (I_{total}) inside the resolution range of 0-5 nm^{-1}.

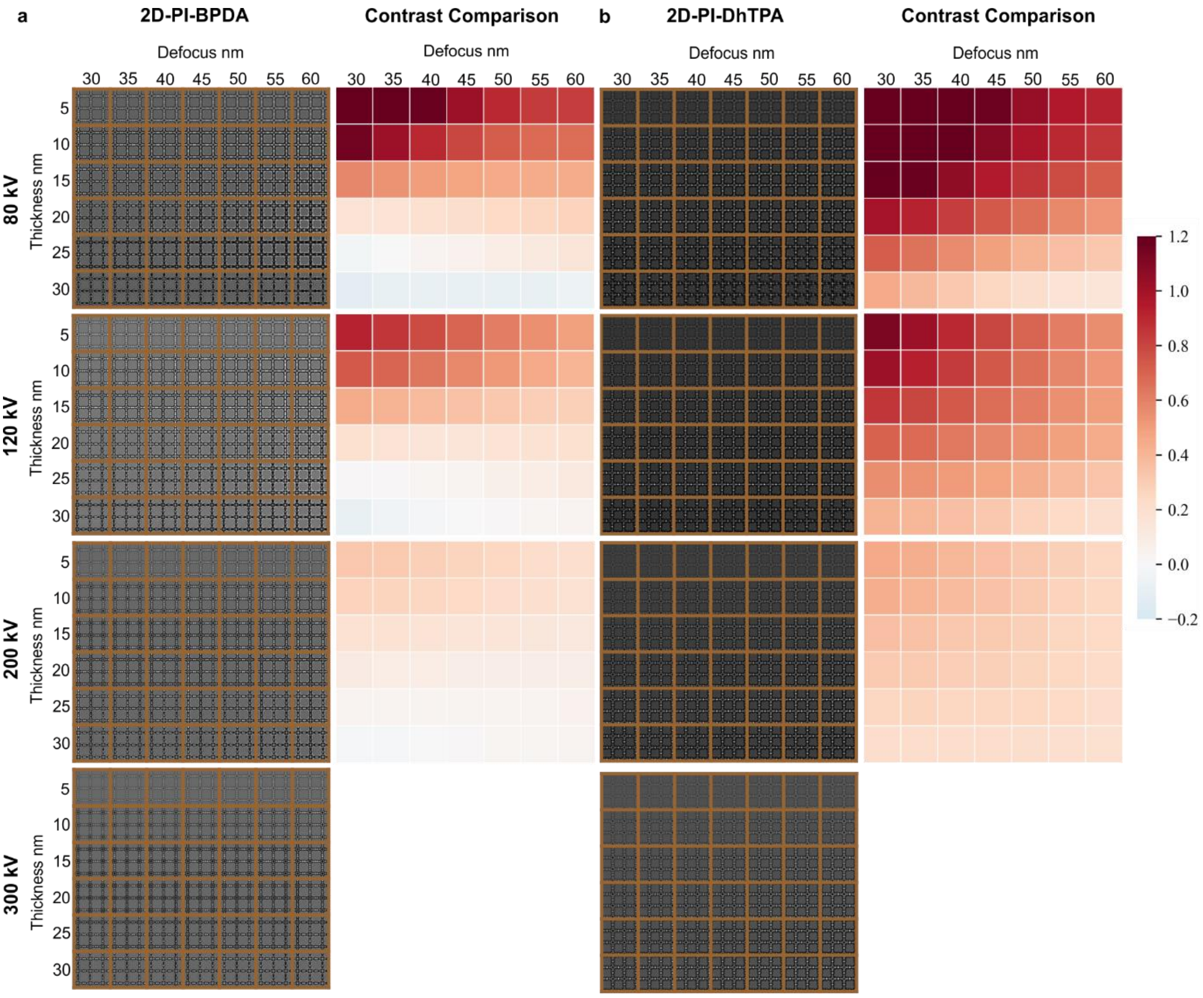

Fig. S6. Contrast comparison of 80, 120, and 200 kV to 300 kV for 2D-PI-BPDA and 2D-PI-DhTPA. The contrast enhancement at 80, 120, and 200 kV is demonstrated via HRTEM image simulation on 2D-PI-BPDA and 2D-PI-DhTPA, in column a and b, respectively. Multislice image calculation has been carried out using the following parameters: pixel size: 0.84 Å; spherical aberration coefficient: -9 µm; chromatic aberration coefficient: 1.4 mm; focal spread: 2 nm, convergence angle: 0.2 mrad; slice thickness: 1 Å; scattering factor: atomic scattering factor. The coefficient of variation of the image grayscale is employed to represent the contrast. The contrast difference between a certain voltage and 300 kV, i.e., ($Contrast_{voltage}$-$Contrast_{300}$)/$Contrast_{300}$, is presented by the heat maps.

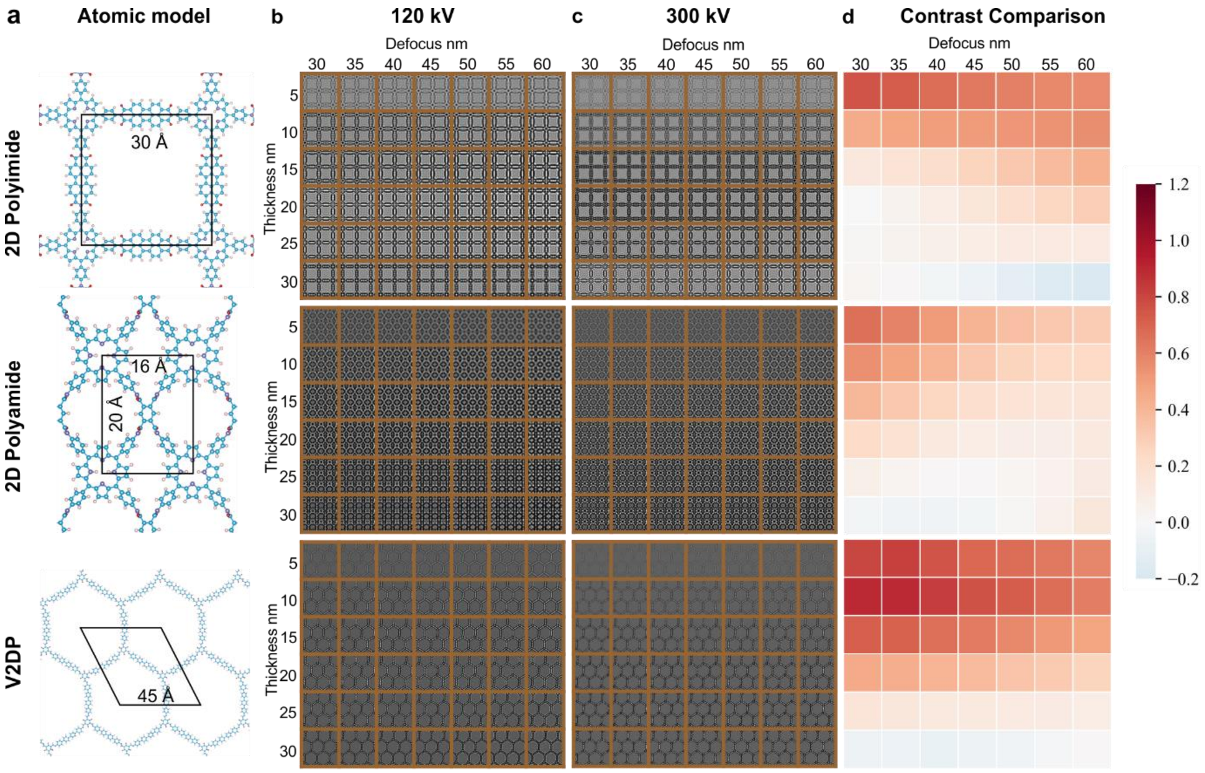

Fig. S7. Contrast comparison between 300 kV and 120 kV. The contrast enhancement at 120 kV is further demonstrated via HRTEM image simulation on 2D polyimide, 2D polyamide, and viologen-immobilized 2DP (V2DP). The atomic models obtained by DFTB calculations are presented in column a. Columns b, c present simulated thickness-defocus maps at 120 kV and 300 kV, respectively. Multislice image calculation has been carried out using the following parameters: electron fluence: 70 e-/Å^2; pixel size: 0.84 Å; spherical aberration coefficient: -9 µm; chromatic aberration coefficient: 1.4 mm; focal spread: 2 nm, convergence angle: 0.2 mrad; slice thickness: 1 Å; scattering factor: atomic scattering factor. The coefficient of variation of the image grayscale is employed to represent the contrast. The contrast difference between 120 kV and 300 kV, i.e., ($Contrast_{120}$-$Contrast_{300}$)/$Contrast_{300}$, is presented by the heat maps in column d.

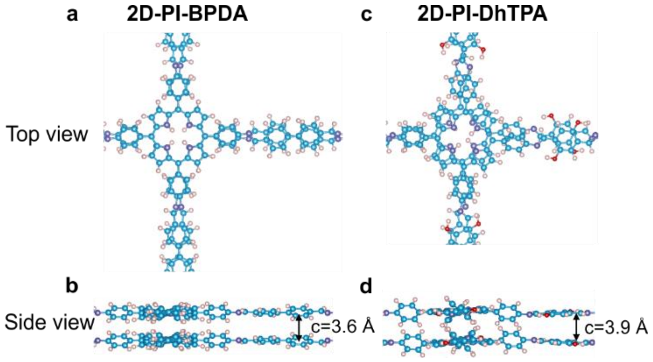

Fig. S8. DFTB calculated structures of 2D-PI-BPDA and 2D-PI-DhTPA. (a and c) top view. (b and d) side view. Both models have monoclinic crystal lattices. The lattice parameters for 2D-PI-BPDA are a=b=30.1 Å, c=3.6 Å, α=90.1°, β=89.8°, γ=90.4°, and for 2D-PI-DhTPA, a=b=25.9 Å, c=3.9 Å, α=84.3°, β=94.2°, γ=89.9°.

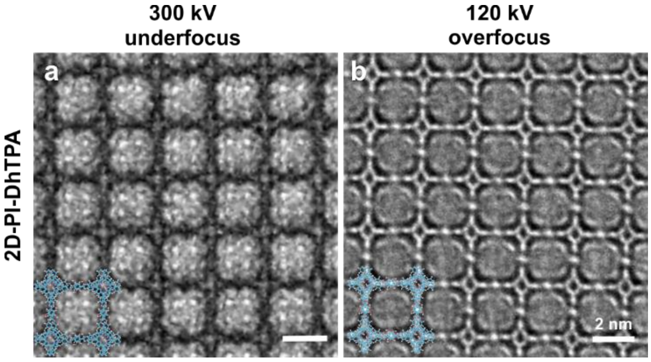

Fig. S9. AC-HRTEM imaging of 2D-PI-DhTPA at 300 kV and 120 kV. a, AC-HRTEM image acquired at 300 kV with underfocus condition (defocus: ca. - 150 nm). The polymer network appears dark on a bright background. b, AC-HRTEM image acquired at 120 kV with overfocus condition (defocus: ca. 50 nm). The polymer network appears bright on a dark background. The pore on TAPP molecules can be clearly resolved.

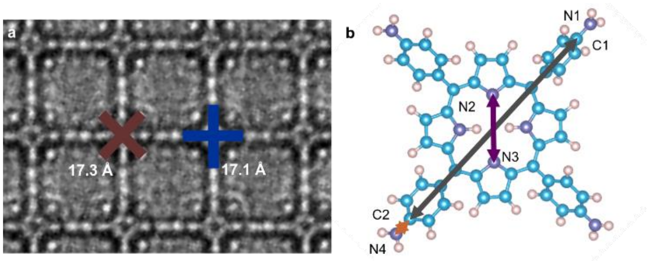

Fig. S10. The scale of the TAPP molecule in AC-HRTEM image and literature. a, AC-HRTEM image of 2D-PI-BPDA. The scale of the TAPP molecule in the polymer network is approximately 17.1 Å. The distance between two diagonal spots at the corner of the framework pore is ca. 17.3 Å. b, Atomic structure of the TAPP molecule. The distance between C1 and C2 is approximately 15.4 Å [1]. The distance of the C-N bond in aniline is 1.4 Å [2], corresponding to the distance between C2 and N4. The distance between N1 and N4 is, therefore, ca. 18.2 Å. According to DFT calculations, the pore size (N2 to N3) of the TAPP molecule is approximately 4.1 Å [3].

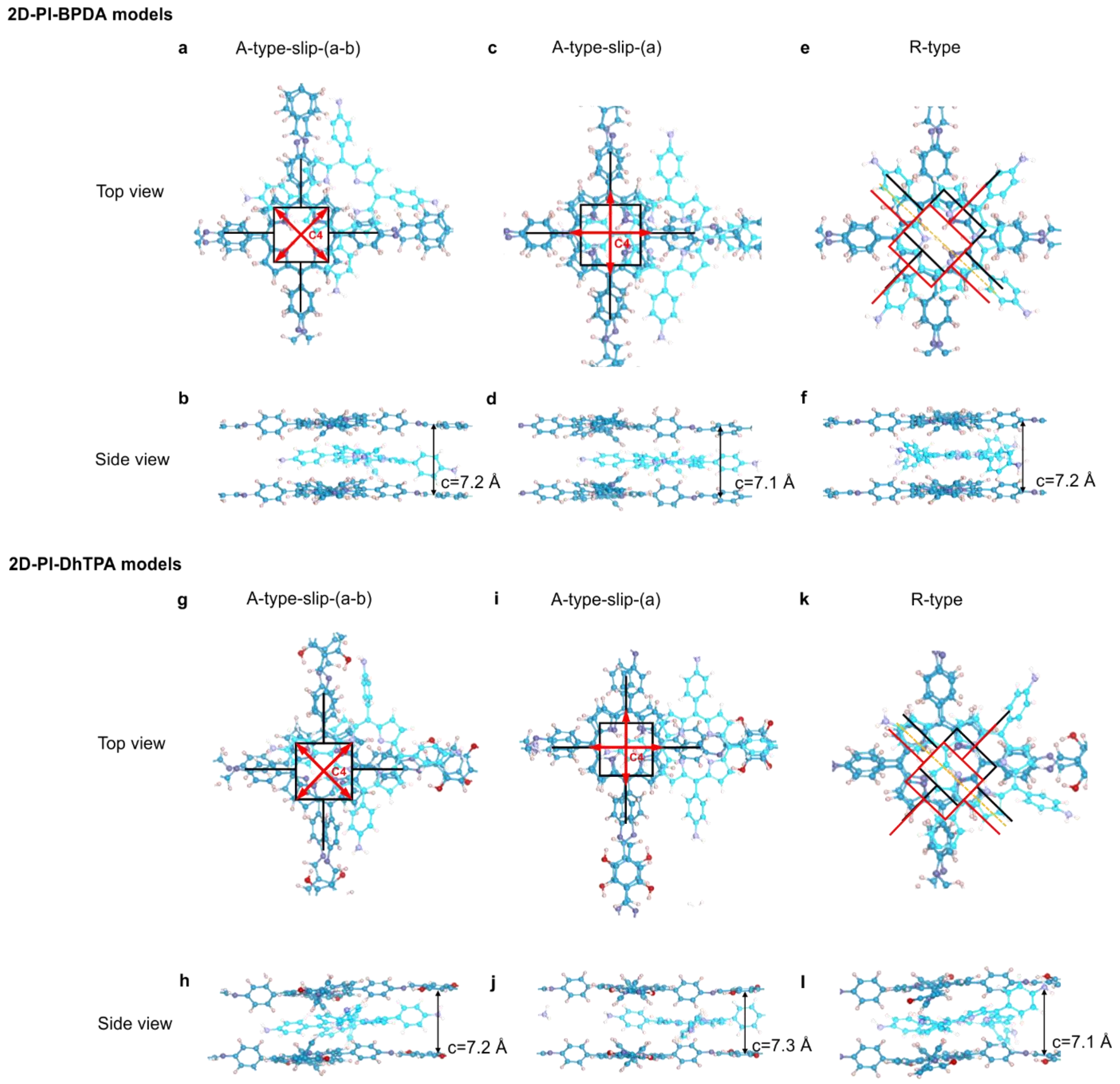

Fig. S11. DFTB calculated 2D-PI-BPDA and 2D-PI-DhTPA double-layer structures with TAPP interstitials. The two A-types and one R-type model of both 2DPs are presented in the top and side view. The distances between 2DP layers are labeled next to the side view of the models.

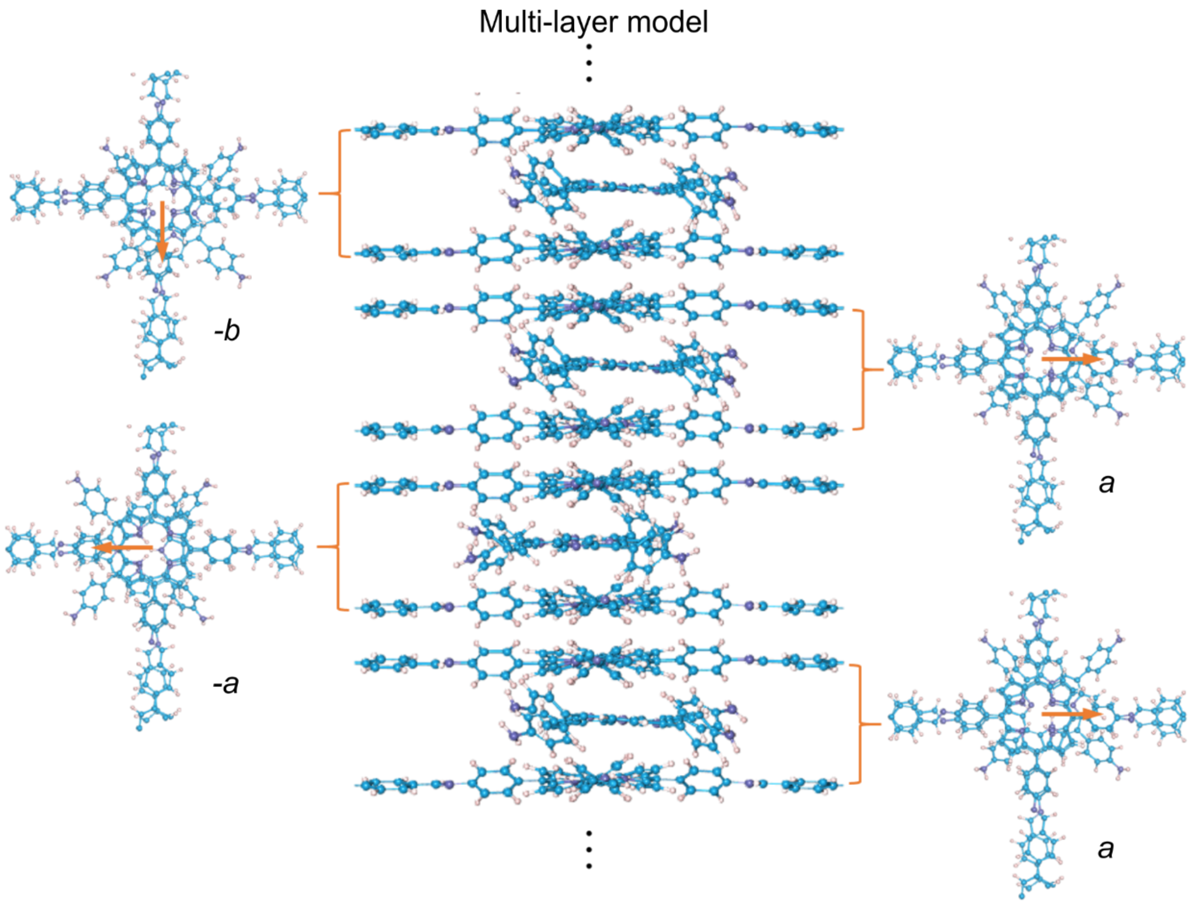

Fig. S12. A schematic representation of the construction of the statistical multi-layer model with TAPP interstitials. As exemplified by the R-type 2D-PI-BPDA, the interstitial TAPP molecules are rotated by 43° with respect to the framework and may shift towards one of the following directions, i.e., *a*, -*a*, *b*, -*b*. Shift directions are nearly isoenergetic, so the multilayer system was built from the four interstitial bilayer structures, with the shift directions randomly distributed. The schematic structure is shown in the middle of the figure. Insets on the sides show the top-view of the particular interstitial TAPP molecules between 2D-PI-BPDA layers.

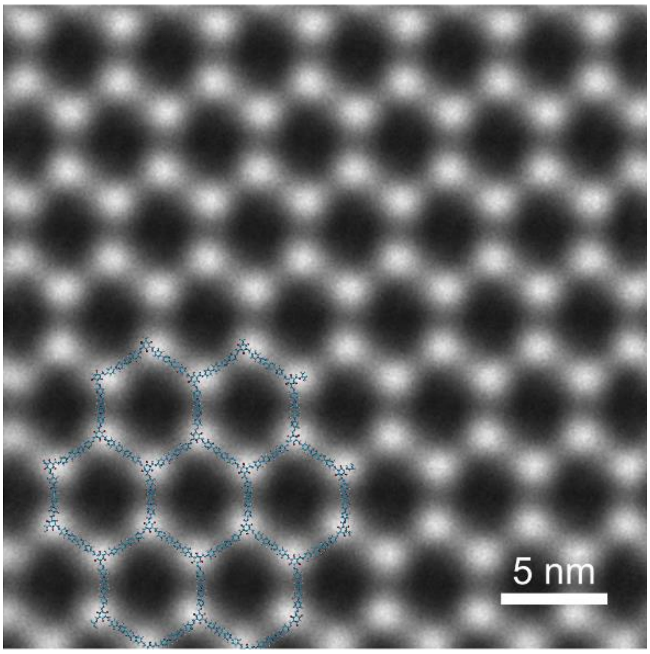

Fig. S13. Image simulation of viologen-immobilized 2DP. Multislice simulation was carried out with the following parameters: acceleration voltage 120 kV, thickness 20 nm, defocus 4.4 µm, electron fluence: 170 $e^-/Å^2$; pixel size: 0.9 Å; spherical aberration coefficient: -9 µm; chromatic aberration coefficient: 1.4 mm; focal spread: 2 nm, convergence angle: 0.2 mrad; slice thickness: 1 Å; scattering factor: atomic scattering factor. The atomic structure is overlaid. Each bright spot in the image represents a node molecule in the framework.

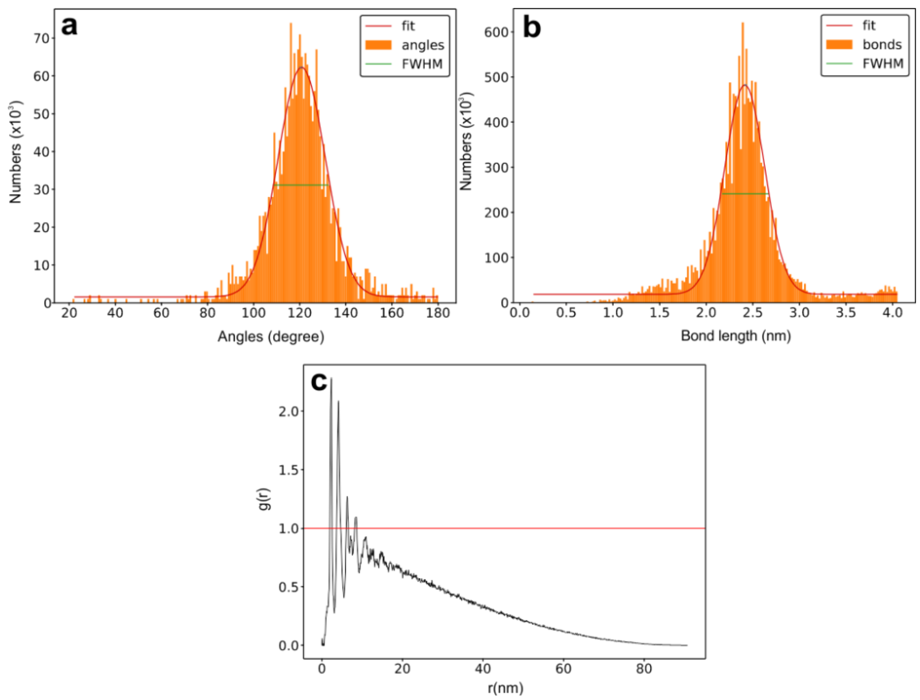

Fig. S14. Statistical analysis of *a*-PI. The statistical analysis results are obtained from a sample area of over 200 nm^2. a, Bond distance distribution. The orange bars are the statistical results of different bond angles, and the red curve is the Gaussian fit of the peak. b, Bond length distribution. The orange bars are the statistical results of different bond angles, and the red curve is the Gaussian fit of the peak. The FWHM and peak maximum are presented in Table S2. c, Radial distribution function. The high peaks at the low radius r indicate the short-range order of the sample. The $g(r)$ value represents the probability of finding one neighboring node molecule at a distance of r, when choosing one node molecule as the reference. It is evaluated as $g(r) = \frac{dn_r}{4\pi r^2 dr\rho}$, dn_r is as function that computes the number of particles within a shell of thickness dr, ρ is the bulk density of the material. The fast degradation of the g value below 1 demonstrates the amorphous nature of *a*-PI.

Table S1 Relative energies per unit cell for TAPP interstitials in 2D-PI-BPDA and 2D-PI-DhTPA bulk structures obtained with DFTB2+D3 (BJ) and 3ob-3-1 parameter set and neutral pH conditions.

E_{rel}: kJ/mol	2D-PI-BPDA	2D-PI-DhTPA
A-type-slip-(*a*)	14.0	0.0
A-type-slip-(*a-b*)	17.2	5.7
R-type	0[a]	14.6

[a] In neutral 2D-PI-BPDA, there is also an AA-eclipsed type interstitial TAPP with E_{rel} of 10 kJ/mol. However, it is improbable to occur in the experiment due to low pH during the polymer synthesis[4].

Table S2 Statistical analysis of *a*-PI

	Expected value	Peak value	FWHM
Molecule distance	2.6 nm	2.5 nm	0.5 nm
Molecule-molecule angle	120°	120.6°	24.0°
Polygon distribution (%)	Pentagon	Hexagon	Heptagon
	22	53	24

References

1. Nurhayati *et al.* Revealing the Real Size of a Porphyrin Molecule with Quantum Confinement Probing via Temperature-Dependent Photoluminescence Spectroscopy. *J. Phys. Chem. A* **124**, 2672–2682 (2020).
2. Zhang, H., Jiang, X., Wu, W. & Mo, Y. Electron conjugation: Versus π-π Repulsion in substituted benzenes: Why the carbon-nitrogen bond in nitrobenzene is longer than in aniline. *Phys. Chem. Chem. Phys.* **18**, 11821–11828 (2016).
3. Marchand, G., Roy, H., Mendive-Tapia, D. & Jacquemin, D. N-confused porphyrin tautomers: lessons from density functional theory. *Phys. Chem. Chem. Phys.* **17**, 5290–5297 (2015).
4. van der Weegen, R., Teunissen, A. J. P. & Meijer, E. W. Directing the Self-Assembly Behaviour of Porphyrin-Based Supramolecular Systems. *Chem. - A Eur. J.* **23**, 3773–3783 (2017).
