# Supplementary material for: Optimal acceleration voltage for near-atomic resolution imaging of layer-stacked 2D polymer thin films
Source: Nat Commun. 2022 Jul 8;13:3948. doi: 10.1038/s41467-022-31688-4 (PMC9270374; doi:10.1038/s41467-022-31688-4)
Supplement: Supplementary file 3 — Source Data [file 41467_2022_31688_MOESM3_ESM.zip › Source data/Readme.docx]

**Readme file for data linked to papar**

‘*Optimal acceleration voltage for near-atomic resolution imaging of layer-stacked 2D polymer thin films’*

By *Baokun Liang, Yingying Zhang, Christopher Leist, Zhaowei Ou, Miroslav Položij, Zhiyong Wang, David Mücke, Renhao Dong, Zhikun Zheng, Thomas Heine, Xinliang Feng, Ute Kaiser, Haoyuan Qi*

The following data are stored in the excel file:

1. The measurrments of critical fluence, structural information and the information coefficient of 2D- PI-BPDA and 2D-PI-DhTPA, the averaged results of the data corredpond to the line plots in Figure 1 d,e,f.
2. The coefficient of variation of the QSTEM simulations of the two above mentioned sample: at 120 kV, 300 kV, and the comparison results, which correspond to the heatmaps in Figure 2.
